# Somatostatinergic systems: an update on brain functions in normal and pathological aging

**DOI:** 10.3389/fendo.2012.00154

**Published:** 2012-12-06

**Authors:** Guillaume Martel, Patrick Dutar, Jacques Epelbaum, Cécile Viollet

**Affiliations:** Inserm UMR894 - Center for Psychiatry and Neuroscience, Université Paris Descartes, Sorbonne Paris CitéParis, France

**Keywords:** interneurons, SRIF, GPCR, sst, cortistatin, long-range, Alzheimer’s disease

## Abstract

Somatostatin is highly expressed in mammalian brain and is involved in many brain functions such as motor activity, sleep, sensory, and cognitive processes. Five somatostatin receptors have been described: sst_1_, sst_2_ (A and B), sst_3_, sst_4_, and sst_5_, all belonging to the G-protein-coupled receptor family. During the recent years, numerous studies contributed to clarify the role of somatostatin systems, especially long-range somatostatinergic interneurons, in several functions they have been previously involved in. New advances have also been made on the alterations of somatostatinergic systems in several brain diseases and on the potential therapeutic target they represent in these pathologies.

## NEUROANATOMY OF SOMATOSTATINERGIC SYSTEM

### SOMATOSTATIN PEPTIDES IN THE BRAIN

Somatostatin_14_ (SRIF, somatotropin release inhibiting factor) was serendipitously discovered in 1972 by Roger Guillemin and colleagues who were aiming to purify and characterize growth hormone (GH)-releasing hormone from sheep hypothalamus (Brazeau et al., 1973). Soon thereafter, an N-terminally extended peptide, SRIF_28_, was purified from the gut. Both peptides, arising from a common propeptide encoded from a single gene, were found in the mammalian nervous system where SRIF_14_ is the predominant form (for review, see Epelbaum, 1986).

**FIGURE 1:**
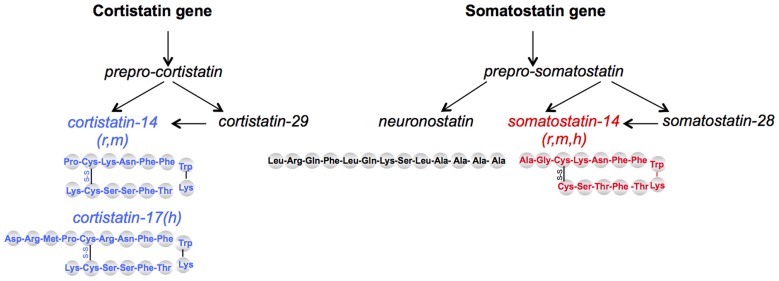
**Schematic representation of somatostatin-related peptides.** SRIF_14_ and CST_14_ come from two distinct genes but bind the five mammalian sst receptors. Neuronostatin is encoded by the same gene as SRIF but does not bind SRIF receptors; its effects seem mediated through the melanocortin system. r, rat; m, mouse; h, human.

Two brain SRIF-related bioactive peptides have been discovered later. Cortistatin (CST) has been cloned in 1996 (De Lecea et al., 1996) and shares 11 amino acids with SRIF (**Figure  1**). CST peptides are predicted to occur as 14-AA or 17-AA short forms in rodents and humans, respectively, and a 29-AA extended form in both species. CST is mainly restricted to the cerebral cortex and the hippocampus in the central nervous system (CNS). CST has been implicated in several brain functions such as learning and memory, regulation of sleep/wakefulness rhythms and it is suspected to have an anticonvulsant activity (for review, see de Lecea, 2008). Recently, bioinformatics analyses of evolutionary conserved sequences identified neuronostatin, a 13-AA amidated peptide also encoded by the somatostatin gene. Mostly found in pancreas, spleen, and brain, it is involved in metabolic, cardiovascular, and neuronal functions (Samson et al., 2008).

Somatostatin induces many transduction mechanisms in transfected systems (for review, see Lahlou et al., 2004; Olias et al., 2004), but deciphering the physiological actions of the native receptors *in situ* remains an intense field of study. The last decade showed increasing progress in understanding the role of SRIF in brain functions using molecular, pharmacological, and behavioral approaches. The development of innovative molecular, genetic, and imaging tools now allows to go a step further and to assess the cellular contribution of SRIF-expressing cells in neuronal networks *ex vivo* and soon *in vivo*. In this review we will give an overview of the latest findings concerning SRIF systems in brain, report some recent data concerning their synaptic actions and their physiological roles within the brain, in normal or pathological conditions.

### SOMATOSTATINERGIC NETWORKS IN THE BRAIN

**FIGURE 2:**
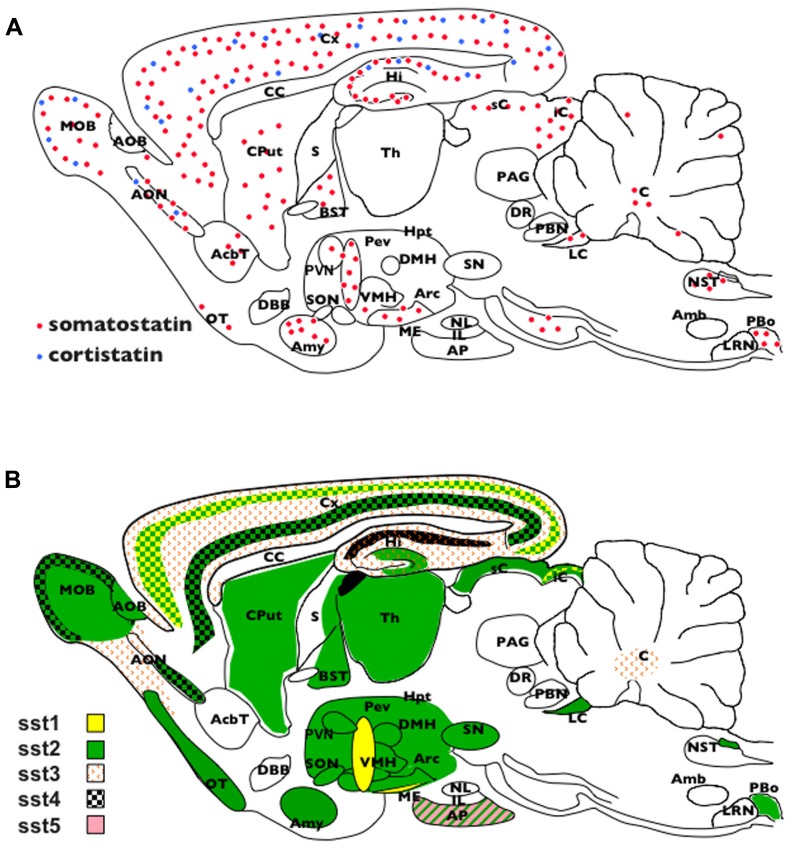
Schematic representation of somatostatin and cortistatin distribution in the mouse brain. **(B)** Schematic representation of somatostatin receptors distribution in the mouse brain. AcbT, nucleus accumbens; Amb, ambiguous nucleus; AP, anterior pituitary; AOB, accessory olfactory bulb; Amy, amygdala; Arc, arcuate nucleus; BNST, bed nucleus of the stria terminalis; CC, corpus callosum; CPut, caudate putamen; Cx, cortex; DBB, diagonal band of broca; DMH, dorsomedian hypothalamus; DR, dorsal raphe; iC, inferior colliculus; IL, intermediate lobe of the pituitary; Hi, hippocampus; Hpt, hypothalamus; LRN, lateral reticularis nucleus; ME, median eminence; MOB, main olfactory bulb; NL, neural lobe of the pituitary; NTS, nucleus of the solitary tract; OT, olfactory tubercle; PAG, periaqueductal gray; PBo, pre-Bötzinger nucleus; PeV, periventricular nucleus; PVN, paraventricular nucleus; sC, superior colliculus; S, septum; SON, supraoptic nucleus; SN, substantia nigra; Th, thalamus; VMH, ventromedian hypothalamus.

Somatostatin is ubiquitously expressed in mammalian brain, including humans (**Figure 2A**). SRIF-immunoreactivity is found at high level in the mediobasal hypothalamus and median eminence, amygdala, preoptic area, hippocampus, striatum, cerebral cortex, sensory regions, and the brainstem (for review, see Epelbaum, 1986; Viollet et al., 2008).

Somatostatin peptide colocalizes with gamma-aminobutyric acid (GABA), a major inhibitory neurotransmitter and labels mostly non-glutamatergic cells in the brain. In order to target SRIF interneurons *in situ*, most recent studies took advantage of rodent models expressing the green fluorescent protein (GFP) under the control of the GAD67 promoter to visualize GABAergic populations more easily. In the GIN (GFP-expressing inhibitory neurons) strain (Oliva et al., 2000) nearly all GFP cells stained for SRIF while in the GAD67-GFP strain, SRIF immunohistochemistry labels 37% of total GFP cells (Ma et al., 2006). The recent development of specific Cre recombinase and knock-in inducible driver lines for SRIF (Taniguchi et al., 2011) opens promising avenues to study SRIF functions at the cellular level combined to optogenetic and imaging tools.

Previous immunohistochemical and tracing studies have identified two main categories of SRIF neurons: those acting locally in a given structure within microcircuits (interneurons) and those projecting to a distant structure (long-projecting neurons). Nevertheless, recent data using GFP transgenic mice revisit previous anatomical records by demonstrating that some formerly called interneurons also belonged to the projecting neurons category. The different kinds of GABAergic interneurons are classified according to their molecular, physiological, and morphological properties. Immunohistochemical characterization of neuronal populations in rat cortex initially stated, based on calcium-binding proteins and peptides expressions that parvalbumin (PV), SRIF, calretinin, and cholecystokinin labeled four main non-overlapping chemical classes of interneurons (Xu et al., 2006). However, several studies later reported a significant colocalization of SRIF and calretinin in mouse brain (Xu et al., 2006; Kosaka and Kosaka, 2007; Lepousez et al., 2010a), pointing out species-dependent variations in the repertoire of calcium-binding proteins and neuropeptides. It seems that neuronal populations immunoreactive for calbindin or the neuropeptide Y strongly overlap with the somatostatinergic population in rats whereas calretinin is preferentially coexpressed with SRIF in the mouse.

Recent morphological and electrophysiological studies using GIN mice focused on SRIF-expressing populations in cortical circuits. In mouse cortex, parvalbumin- and SRIF-expressing neurons respectively constitute 40 and 30% of the total GABAergic neurons, calretinin being expressed in 50% of the somatostatinergic population (Rudy et al., 2011). The remaining cortical inhibitory interneurons, expressing ionotropic serotonergic receptor 5HT3a, include VIP- and NPY-positive subpopulations whose partial colocalization with SRIF has been reported (Gonchar et al., 2007; Xu et al., 2010; Rudy et al., 2011). SRIF-positive interneurons are homogeneously distributed in all cortical layers (2–6), as compared to PV-positive inhibitory interneurons that are concentrated in the upper part of the layer (Perrenoud et al., 2012).

The major class of SRIF interneurons, the Martinotti cells, have ascending axons that arborize and spread horizontally in layer 1, targeting the distal dendritic parts of excitatory pyramidal neurons (for review, see Viollet et al., 2008). Excitatory inputs onto Martinotti cells are generally strongly facilitating, allowing feedback inhibition of the excited pyramidal cell that increases as function of the rate and the duration of the presynaptic discharge (Kapfer et al., 2007; Silberberg and Markram, 2007). The relative distance between excitatory and interneurons inputs may also impact feedback selectivity and grade, inhibition being stronger for closer inputs. A recent study using a two-photon microscopy approach coupled to uncaged glutamate in cortical slices of GIN mouse mapped the inhibitory network between SRIF-positive interneurons and pyramidal cells at the single-cell resolution (Fino and Yuste, 2011). Whatever the pyramidal cell stimulated, it led to a dense innervation of the surrounding somatostatinergic interneurons, with activity related to the proximity of the cells. Notably, this inhibitory connectivity looked unspecific as all inhibitory interneurons were locally connected to every sampled pyramidal cells regardless whether these were connected among themselves or not. This dense circuit and the fact that somatostatinergic neurons electrically communicate via gap junctions (Ma et al., 2006; Hu et al., 2011) favors the hypothesis that the entire somatostatinergic population belongs to a same inhibitory cortical circuit, contradicting the hypothesis of specific inhibitory cortical subnetworks.

Additional classes of cortical SRIF inhibitory interneurons have been recently described according to their localization, intrinsic firing properties, expression of molecular markers, and connectivity (Gonchar et al., 2007; McGarry et al., 2010). On one hand, calretinin expression was proposed as a distinctive marker (Rudy et al., 2011), since its expression is associated to distinct neuronal morphology and connectivity in populations with distinct ontogenic origin (Sousa et al., 2009; Xu et al., 2010). On the other hand, two novel SRIF-positive subtypes were identified after cluster and principal component analysis of a whole range of morphological or electrophysiological parameters (McGarry et al., 2010). These cell types have some similarities to neurons labeled in a GABAergic-GFP strain distinct from GIN (X94 strain; Ma et al., 2006), such as the lack of expression in the layer 1, but they target different cortical layers. Future identification of their respective calcium-binding proteins and neuropeptides repertoire as well as their molecular phenotype will help to conciliate these independent classifications based on morphological and electrophysiological properties.

Somatostatin is found in most sensory systems, i.e., retina (Thermos, 2003; Cervia and Bagnoli, 2007). In the olfactory system, SRIF expression has also been described in sparse short-axon cells scattered in the deep part of the granule cell layer (the main site of intrinsic inhibitory neurons; Shipley and Ennis, 1996; Eyre et al., 2009) and in the peripheral glomerular layer (which receives sensory inputs) in some species (Hwang et al., 2004). Recently, a novel type of somatostatinergic interneurons has been described as predominant in the murine olfactory bulb and specific to this species (Lepousez et al., 2010a). SRIF-positive somata and dendritic fields are restricted to the layer of the olfactory bulb where intrinsic GABAergic interneurons and bulbar principal cells interact through dendrodendritic reciprocal synapses to initiate local gamma oscillations responsible for odor processing. Electron microscopy evidences suggest that SRIF-positive interneurons also establish reciprocal dendrodendritic synapses with the bulbar principal cells (mitral cells). SRIF-positive neurons have also been described downstream in the olfactory pathway; SRIF interneurons constitute a major GABAergic population in the pars principalis of the anterior olfactory nucleus and in the olfactory tubercle (Brunjes et al., 2011). In both piriform and entorhinal cortices, two cortical structures involved in the processing of odor coding, multipolar SRIF-positive interneurons displaying Martinotti-like morphological and electrical properties are found in the deep (Young and Sun, 2009; Saiz-Sanchez et al., 2010; Suzuki and Bekkers, 2010) and superficial (Saiz-Sanchez et al., 2010; Tahvildari et al., 2012) layers respectively.

**FIGURE 3:**
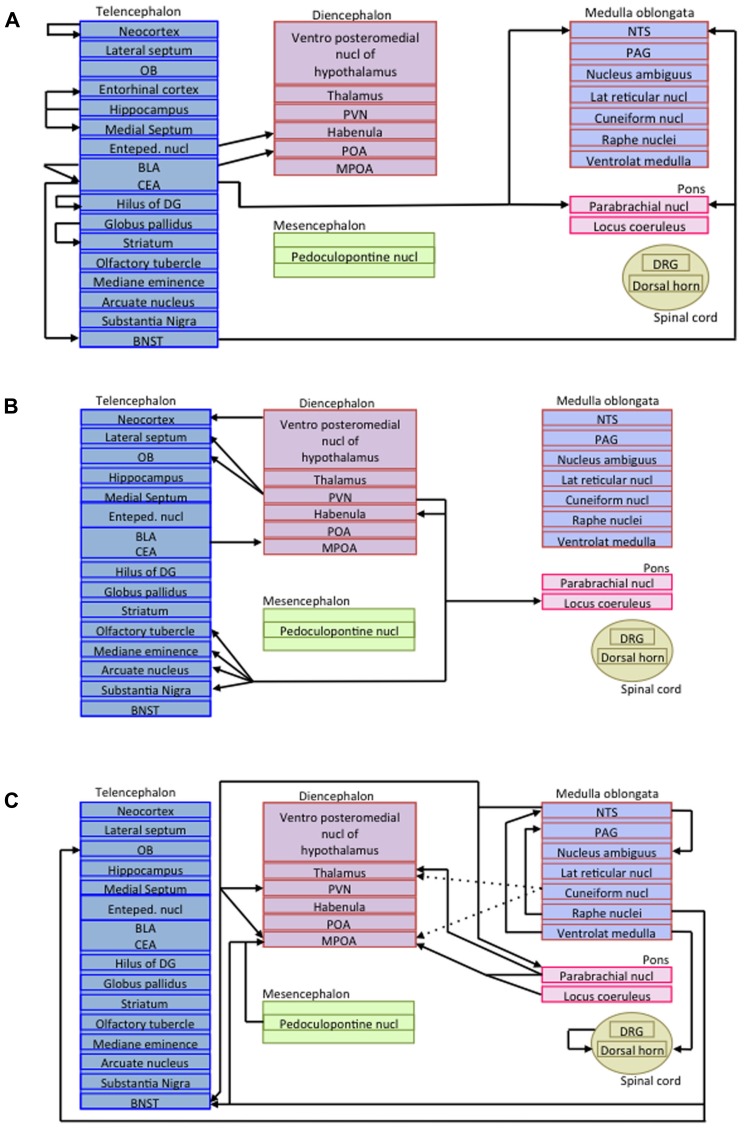
**Schematic representations of long-range somatostatinergic interneurons in the central nervous system.** **(A)** Telencephalic efferent projections to the rest of the brain. **(B)** Efferent projections arising from the diencephalon and projecting to the telencephalon and the pons. **(C)** Efferent projections arising from the mesencephalon, the medulla oblongata, the pons and the spinal cord. Enteped, entopeduncular; BLA, basolateral amygdala; BNST, bed nucleus of the stria terminalis; CEA, central amygdala; DG, dentate gyrus; DRG, dorsal root ganglia; MPOA, medial preoptic area; NTS, nucleus of the solitary tract; OB, olfactory bulb; PAG, periaqueductal gray; POA, preoptic area; PVN, paraventricular nucleus.

As mentioned before, in addition to the somatostatinergic interneurons acting within microcircuits, long-projecting somatostatinergic cells have been described in several regions (Viollet et al., 2008). As glutamatergic pyramidal cells projections do, long-range inhibitory connections mediate communication between multiple brain areas. Long-range inhibitory terminals have a larger diameter and a thicker myelin layer than excitatory projection neurons, suggesting that inhibitory signal may precede the arrival of excitation in co-innervated cortical areas (Jinno et al., 2007). Long-range projecting SRIF-containing neurons are encountered in numerous brain areas (for review, see Viollet et al., 2008) such as the hippocampus (Jinno et al., 2007), the cerebral cortex (Tomioka et al., 2005), and the amygdala (McDonald et al., 2012). For instance, virtually all non-pyramidal neurons in the amygdala that have long-range projecting axons to the basal forebrain in the rat express SRIF (McDonald et al., 2012). **Figure 3** represents the projections of all long-range somatostatinergic interneurons known to date in the brain.

### SOMATOSTATIN RECEPTORS IN THE CENTRAL NERVOUS SYSTEM

Autoradiographic studies characterized initially two SRIF binding site according to their affinity for the synthetic agonist octreotide and their pattern of expression. In the early 1990s, five receptors (sst_1__-__5_) belonging to the G-protein-coupled receptors (GPCRs) family were cloned and characterized from various species. Sequence homology is 39–57% among the five subtypes, each being highly conserved across species. They activate multiple intracellular targets (Olias et al., 2004) and display distinct internalization and dimerization properties (Csaba et al., 2012). Based on structural, pharmacological, and operational features, they are now divided into two groups displaying nanomolar affinity for both SRIF and CST: SRIF-1 (sst_2_, sst_3_, and sst_5_ receptors) and SRIF-2 (sst_1_ and sst_4_ receptors). Figure 2B represents the wide expression of SRIF receptors in the CNS. In contrast to most GPCRs, sst_1-5_ are unique because their gene coding sequence is devoid of introns. However, this does not preclude the generation of spliced variants such as a shorter isoform of mouse sst_2_, named sst_2B_, originating by the excision of a cryptic intronic sequence (Vanetti et al., 1992), and spliced variants of sst_5_ in human and rodents (Córdoba-Chacón et al., 2011). While some data suggested that CST also acts through the proadrenomedullin receptor MgrX2 or the ghrelin orexigenic peptide receptors, the existence of specific CST receptors has not been demonstrated (Siehler et al., 2008). Neuronostatin does not bind to SRIF receptors, but some of its effects seem mediated through the central melanocortin system (Yosten et al., 2011). Recent findings have shown that neuronostatin is involved in regulating depressive behavior and nociception (Yang et al., 2011a,b, 2012).

The extended distribution of sst_2_ receptors in the CNS together with studies using subtype selective SRIF analogs in both *in vivo* and *in vitro* experiments, suggested that these subtypes are the major players in the SRIF receptor family. They have broad inhibitory effects in many neuronal networks including cortex, hippocampus, limbic regions, and sensory systems (retina and olfactory system; Viollet et al., 2008; Lepousez et al., 2010a; Radojevic et al., 2011). The sst_1_ receptor may function as an autoreceptor in basal ganglia, hypothalamus, and sensory systems (Thermos et al., 2006), and in the hippocampus (de Bundel et al., 2010). sst_3_ receptors are localized to mature neuronal cilia in most brain regions (Stanić et al., 2008), and pharmacological or genetic blockade of sst_3_ have marked behavioral effects (Einstein et al., 2010). sst_4_ receptors are highly expressed in the olfactory bulb, cortex, and hippocampus, where their role remains to be clarified. In the mouse they modulate epileptic activity, whereas in the rat it seems that this effect is largely related to sst_2_ receptors. Hippocampal sst_4_ have also been involved in cognitive processes (Gastambide et al., 2009; Sandoval et al., 2011), functionally interacting with sst_2_ (Dutar et al., 2002; Gastambide et al., 2010). sst_5_ receptors mediate regulation of GH release and inhibit cell proliferation by SRIF/CST, mainly through sst_2_/sst_5_ receptors interaction. The detection of functional truncated forms of sst_5_ suggests that they could interfere in and modulate those interactions (Córdoba-Chacón et al., 2011).

## SOMATOSTATINERGIC FUNCTIONS IN THE BRAIN

### NEURONAL ACTIONS OF SRIF

#### Presynaptic Mechanisms

Somatostatin, like other neuropeptides, can modulate CNS excitability via presynaptic mechanisms (Baraban and Tallent, 2004). In rat hippocampus and cortex, SRIF induces a presynaptic inhibition of excitatory neurotransmission leading to a decrease in glutamate release and in the amplitude of evoked synaptic responses (Ishibashi and Akaike, 1995; Boehm and Betz, 1997; Tallent and Siggins, 1997; Grilli et al., 2004). The SRIF-induced decrease in glutamate release is explained by an inhibition of excitatory transmission via a G-protein of the Gi/Go family and modulation of calcium channels. Indeed, SRIF selectively inhibits N-type Ca^2+^ channel via the picrotoxin-sensitive G(i)/G(o) protein. Somatostatin can also inhibit N-type Ca^2+^ channels in the dentate gyrus (Baratta et al., 2002). By these inhibitory effects on excitatory synaptic transmission, SRIF, co-released with GABA on dendritic shafts of principal neurons, increases and prolongs GABA effect. This presynaptic action on Ca^2+^ conductance could explain, at least in part, the inhibitory effect of SRIF on long-term potentiation in the mouse dentate gyrus (Baratta et al., 2002). Other studies suggest that presynaptic K^+^ channels modulation may also be involved in the SRIF inhibition of excitatory transmission (Tallent and Siggins, 1997). More precisions on the mechanisms have been given by Grilli et al. (2004), demonstrating on synaptosomal preparations from mouse cerebral cortex that activation of sst_2_ presynaptic receptors may inhibit the cAMP/PKA pathway stimulated by high potassium concentration, leading to a decrease of the evoked glutamate release. If in the hippocampus, cortex and also hypothalamus, the presynaptic effects of SRIF concern almost exclusively the excitatory transmission (Peineau et al., 2003), SRIF is also able to decrease GABA release in different brain structures, such as the rat basal forebrain (Momiyama and Zaborszky, 2006), the neostriatum (Lopez-Huerta et al., 2008), and the thalamus (Leresche et al., 2000). In the basal forebrain, SRIF presynaptically inhibits both GABA and glutamate release onto cholinergic neurons in a Ca^2+^-dependent way.

#### Postsynaptic Mechanisms

Effects of SRIF on intrinsic neuronal membrane properties are well documented. Somatostatin induces a membrane hyperpolarization resulting from the activation of two distinct K^+^ current, the voltage-sensitive K^+^ current or M-current (*I*_M_; Moore et al., 1988; Jiang et al., 2003), and a voltage-insensitive leak current (Schweitzer et al., 1998). In hippocampal CA1 pyramidal neurons, sst_4_ seems to be the receptor subtype that couples to *I*_M_ (Qiu et al., 2008).

In medium spiny neostriatal neurons, SRIF produces a qualitative change in the firing pattern from a tonic regular to an interrupted “stuttering”-like pattern (Galarraga et al., 2007). These authors demonstrated that SRIF changes the firing pattern via sst_2_-subtype activation, which reduces the small conductance Ca^2+^-activated K^+^ currents (SK-channels) and activates large conductance g(K)Ca^2+^ (GK channels). These results highlight the fact that SRIF is a regulator of cellular function in the striatum. The numerous effects of SRIF on Ca^2+^ and K^+^ channel conductance in different structures are reviewed by Cervia and Bagnoli (2007).

A huge amount of literature has tried to define the pharmacological nature of SRIF effects, using agonists and antagonists of SRIF receptors or mice invalidated for receptor subtypes. Results are often controversial and are different in mice and rats (Aourz et al., 2011). Therefore, the classification of SRIF effects is complex and it is accentuated by the description of functional cooperation between different receptor subtypes sst_2_/sst_3_, sst_2_/sst_4_, sst_3_/sst_4_ (Moneta et al., 2002; Gastambide et al., 2010; Aourz et al., 2011). Recent publications suggest that sst_3_ and sst_4_ (but not sst_1_; de Bundel et al., 2010) have potent anticonvulsive properties (Aourz et al., 2011), and that sst_2_, the major receptor subtype involved in the anticonvulsant effect of SRIF in the hippocampus exerts a functional cooperation with sst_3_/sst_4_. In hippocampus, sst_1_ activation inhibits both NMDA- and AMPA-mediated responses but did not affect the inhibitory transmission (Cammalleri et al., 2009).

### SRIF-CONTAINING NEURONS ARE INVOLVED IN PHYSIOLOGICAL FUNCTIONS

#### Interneurons

A large diversity of inhibitory interneurons is able to exert inhibition on specific compartments of principal cells. Among these populations is the dendrite-targeting SRIF-expressing interneuron located in oriens-lacunosum moleculare of the hippocampus. These SRIF-containing neurons are the only subtype of interneuron that reliably follows synaptic stimulation of the alveus in the theta frequency range via activation of their kainate receptors, suggesting that they play an important role in theta band frequency oscillations (Goldin et al., 2007). Spontaneous activities of inhibitory interneurons have been characterized and SRIF-containing neurons are described in the cortex and piriform cortex as regular-spiking (Kawaguchi and Kubota, 1998; Suzuki and Bekkers, 2010) or low-threshold spiking neurons (Goldberg et al., 2004), often opposed to the fast spiking PV-containing neurons. In the hippocampus, SRIF neurons are locked to the ascending phase of the theta cycle. However, using an optogenetic inhibition of different populations of interneurons, it was recently demonstrated that silencing SRIF interneurons increases burst firing of pyramidal cells without altering the theta phase of spikes (Royer et al., 2012). Applying optogenetic technique to animals trained to run head-fixed on a treadmill belt rich with visual-tactile stimuli, these authors provided evidence that the dendritic (but not somatic) inhibition of pyramidal neurons by SRIF interneurons is critical for controlling spike burst firing during active exploration. They concluded that perisomatic PV-targeting interneurons control the spikes’ theta phase while the dendrite-targeting SRIF interneurons control the rate of discharge. This is in agreement with the fact that dendritic but not somatic GABAergic inhibition is decreased in experimental epilepsy (Cossart et al., 2001). Combining optogenetic stimulation with *in vivo* two-photon imaging in the mouse visual cortex, Wilson et al. (2012) demonstrate that soma-targeting PV neurons regulate the gain of cortical response, while dendritic-targeting SRIF neurons shift response level and alter stimulus selectivity, leaving response gain unaffected.

Another demonstration of the role of SRIF interneurons in cellular function has been given recently (Gentet et al., 2012). In this study, SRIF neurons recorded in the barrel cortex of awake mice were tonically active during quiet wakefulness but they decreased their firing during whisker sensorimotor processing. This decrease in firing relieves the dendrites of excitatory pyramidal neurons from inhibition.

It is known that inhibitory neurons have diverse roles in physiological and synaptic function, based on their connectivity patterns and intrinsic properties. All the experiments described above demonstrated that SRIF interneurons have a prominent role in the regulation of distal dendrites excitability.

#### Long-range Projecting Neurons

The long-range projecting somatostatinergic non-pyramidal cells found in the hippocampus target the medial septum and the medial entorhinal cortex (Viollet et al., 2008; Melzer et al., 2012) and more specifically form inhibitory synapses on GABAergic interneurons of these areas. They coordinate activity between distant brain regions, contributing to the generation and the synchronization of rhythmic oscillatory activity in the hippocampus and entorhinal cortex (Melzer et al., 2012). They are therefore involved in spatial and temporal coding. Interestingly, early-generated GABA-containing hub neurons, dendrite-targeting interneurons, express preferentially SRIF and give long-range projecting neurons (Picardo et al., 2011). These superconnected hub cells are present early in the developing hippocampus. They develop a widespread axonal arborization and remain into adulthood. They play a key role in the control of the hippocampal giant depolarizing potentials as well as in the modulation of network dynamics. In the other brain areas, the precise contribution of these long-projecting SRIF neurons in the oscillatory activity still needs to be addressed.

#### Hypophysiotropic Neurons

Somatotropin release inhibiting factor was initially discovered as a neurohormone that inhibits GH secretion from anterior pituitary somatotroph cells. This function is exerted by hypophysiotropic neurons, located in the anterior periventricular hypothalamic nucleus, which project to the median eminence and release the peptide in the fenestrated capillaries of the hypothalamo–hypophyseal portal vessels; thus directly connecting the brain to the anterior pituitary. SRIF is also a potent inhibitor of many hormonal and exocrine secretions as well as an antiproliferative agent in normal and tumoral tissue (Epelbaum, 1986). SRIF analogs (octreotide and lanreotide) have potent inhibitory effects on hypersecretion, thereby alleviating the symptoms associated with neuroendocrine tumors. Furthermore, the antitumor potential of octreotide is now well documented. Pasireotide, a long-acting SRIF analog, has the advantage of targeting a wider range of SRIF receptors (subtypes 1, 2, 3, and 5) than the analogs previously used in clinical practice (which preferentially target subtype 2) and has a broader spectrum of activity (for review, see Bousquet et al., 2012).

### INVOLVEMENT OF SRIF SYSTEMS IN SENSORY, MOTOR, AND COGNITIVE FUNCTIONS

Since SRIF systems are widely expressed in CNS, they are involved in numerous functions including nociceptive and vasoconstrictor properties. Here, we will present recent advances about the role of SRIF systems in autonomic responses (digestion, cardiac rate, and respiration) and motor functions as well as cognitive functions such as learning and memory and emotion (for review, see Viollet et al., 2008).

#### Somatostatinergic Involvement in Sensory Functions

***Somatostatin and visual information*.** Somatostatinergic system is expressed in mammalian retina (for review, see Thermos, 2003; Casini et al., 2005; Cervia et al., 2008), where it is suspected to exert multiple actions on neurons and on retinal physiology. SRIF acts as a positive factor in the retina by regulating homeostasis and protecting neurons against damage. Both sst_2_ and sst_5_ somatostatinergic receptors are involved. Indeed, activation of sst_2_ protects the retina from ischemic insults *ex vivo* (Mastrodimou et al., 2005) and sst_2_ as well as sst_5_ receptor activation protect from excitotoxicity *in vivo* (Kiagiadaki and Thermos, 2008; Kiagiadaki et al., 2010; Kokona et al., 2012). The severity of angiogenic responses to hypoxia is correlated to the sst_2_ expression level in the retina (Dal Monte et al., 2007). Moreover, the sst_2_-preferring agonist octreotide prevents hypoxia-induced VEGF up-regulation (Dal Monte et al., 2009).

***Somatostatinergic modulation of olfactory discrimination*.** Recent studies have shown that SRIF modulates olfactory processing in mice (Lepousez et al., 2010a,b). In mouse main olfactory bulb, SRIF is mainly concentrated in local GABAergic interneurons synaptically connected to the mitral cells by reciprocal dendrodendritic synapses. When activated by an odor, mitral cells synchronize and generate gamma oscillations of the local field potentials that are involved in olfactory processing. Pharmacological or genetic blockade of sst_2_ transmission in the olfactory bulb of awake animal selectively decreased the gamma oscillations power while pharmacological activation of sst_2_ had opposite effects. These treatments were respectively correlated to either impairment or improvement of odor discrimination performances of the pharmacologically injected animals. Thus, bulbar endogenous SRIF, presumably released from external plexiform layer interneurons, affects gamma oscillations through the dendrodendritic reciprocal synapse and contributes to olfactory processing.

#### Involvement of SRIF in Learning and Memory

It has been reported for decades that SRIF plays a role in learning and memory at different stages of information processing. The first studies investigating its role in cognition showed that intracerebroventricular administrations of SRIF improved learning in active avoidance tasks (Bollok et al., 1983; Vecsei et al., 1983; Vecsei and Widerlov, 1988) and prevented electroshock-induced amnesia in passive avoidance paradigms (Vecsei et al., 1983, 1984). Conversely, the depletion of SRIF in the brain by cysteamine (which depletes SRIF levels; Szabo and Reichlin, 1981) produced major memory deficits in passive avoidance (Bakhit and Swerdlow, 1986; Schettini et al., 1988; DeNoble et al., 1989). These studies revealed that SRIF is involved in the acquisition of information but other studies showed that cysteamine produced memory deficits not only when given before the training session but also within a critical time window (0–4 h) after acquisition, suggesting that SRIF plays a critical role in memory consolidation proces-sing (Haroutunian et al., 1987, 1989; Schettini et al., 1988; Vecsei et al., 1990).

The hippocampus is an essential structure in learning and memory (Jeneson and Squire, 2012), and is also a chosen site to study the effects of SRIF on learning and memory since injection of cysteamine impairs tasks requiring its integrity (DeNoble et al., 1989; Guillou et al., 1998). Surprisingly in the rodent hippocampus, both activation of SRIF receptors as well as depletion of SRIF contents generate hippocampal memory impairments. Indeed, microinjections of cysteamine, SRIF or CST directly into the hippocampus impaired hippocampal-dependent spatial learning (Guillou et al., 1993; Sanchez-Alavez et al., 2000; Lamirault et al., 2001; Mendez-Diaz et al., 2005; Gastambide et al., 2009). Consistent with these pharmacological results, transgenic mice overexpressing CST display a profound impairment of spatial learning (Tallent et al., 2005). Studies that investigated which SRIF receptor mediates SRIF memory effect showed that intra-hippocampal injections of the sst_4_ agonist, but not sst_1_, sst_2_, or sst_3_ agonists, dramatically impaired spatial memory formation (Gastambide et al., 2009). Importantly, these authors found that concomitantly to the impairment of spatial memory, an sst_4_ agonist also enhanced the use of striatum-dependent memory. Therefore, it was hypothesized that hippocampal sst_4_ controls the use of cognitive strategies by switching from hippocampus-based multiple associations to simple striatum-based behavioral response through a functional interaction with sst_2_ receptor (Gastambide et al., 2010). The precise cellular and molecular mechanisms involved in this functional interaction between sst_2_ and sst_4_ are not fully understood but some studies showed that sst_4_ mediates increases in glutamatergic excitability and bursting frequency, which were blocked by sst_2_ agonists or antagonists and were lacking in sst_2_ knockout (KO) mice (Moneta et al., 2002; Cammalleri et al., 2006). Therefore, sst_4_ is not the unique SRIF receptor in the hippocampus mediating SRIF memory effects as sst_2_ also modulates memory as previously suggested by Dutar et al. (2002).

#### Involvement of SRIF in the Control of Emotion

Somatostatin and its receptors are strongly expressed in the different nuclei of the amygdala (Hannon et al., 2002), a key brain structure involved in the emotional assessment of the environment (Schumann et al., 2011). Despite the extensive expression of SRIF systems in this area, the effects of SRIF on emotions have not yet been studied extensively. Nevertheless, some studies reported an involvement of SRIF systems in the control of emotion and anxiety. Indeed, a very recent work revealed that the pattern of activation of SRIF-positive interneurons was specific to the nuclei of the amygdala considered and also to the kind of stressor used (Butler et al., 2012). Moreover, SRIF has anxiolytic- and antidepressant-like effects (Engin et al., 2008b) that are associated with the suppression of the frequency of hippocampal theta activity, a neurophysiological signature common to most classes of anxiolytic drugs (i.e., benzodiazepines, selective 5-HT reuptake inhibitors, 5-HT1A agonists). These effects seem to be mediated by sst_2_ receptor since both intra-septal and intra-amygdala SRIF microinfusions induced anxiolytic effects that were completely reversed by selective sst_2_ receptor antagonist injection in these brain areas (Yeung and Treit, 2012). Additional evidence for a specific role of sst_2_ receptor came from the observation that a stressful experience is associated with an increase of sst_2_ mRNA levels within the amygdala (Nanda et al., 2008) and that mice lacking sst_2_ receptor display increased anxiety-like behaviors associated with increased pituitary ACTH levels, a main regulator of the stress response (Viollet et al., 2000).

#### Involvement of SRIF in Locomotion

An involvement for SRIF was also reported in motor functions. Increased motor activity was shown in rats receiving intracerebroventricular administration of SRIF (Havlicek et al., 1976) as well as in mice receiving unilateral striatal infusions of the peptide by retrodialysis (Hathway et al., 2004) and in animals receiving direct injections of SRIF in the nucleus accumbens (Raynor et al., 1993). Tashev et al. (2001) showed that SRIF modulated locomotor activity in biphasic manner. Indeed, shortly after SRIF striatal injection a decrease of locomotor activity is observed whereas later, the locomotor behavior is increased. Similar effects have been found after striatal injection of sst_2_ and sst_4_ agonists. On the other hand, genetic invalidation of sst_2_ receptor in two different strains of mice as well as SRIF null mice showed an impairment of motor functions (Viollet et al., 2000; Zeyda et al., 2001; Allen et al., 2003). But the role of SRIF in locomotion seems to be limited to fine motor control since these different lines of transgenic mice only develop impaired motor coordination in tasks that require a fine motor control and display normal levels of motor activity and coordination in undemanding tasks (Viollet et al., 2000; Zeyda et al., 2001; Allen et al., 2003).

#### Autonomic Responses

Somatotropin release inhibiting factor and its receptors are found in several medulla oblongata nuclei that control autonomic functions such as digestion, cardiac rate, and respiration (Llona and Eugenín, 2005; Spary et al., 2008; Viollet et al., 2008). In the preBötzinger complex (preBötC), a critical component of the respiratory rhythm generator that underlies mammalian breathing, SRIF is expressed in a subpopulation of glutamatergic neurokinin 1 receptor-positive neurons, a kind of neuron rhythmically active (Stornetta et al., 2003). Originating from the homeogene Dbx1 lineage, these cells are mandatory for breathing, since invalidation of the Dbx1 gene impaired their differentiation and disrupted respiratory rhythm generation in the preBötC (Bouvier et al., 2010; Gray et al., 2010). Acute silencing of somatostatinergic preBötC neurons increased respiratory rhythm, leading to persistent apnea (Tan et al., 2008). Similar effects were found *in vitro* after pharmacological blockade of sst_2_ transmission, while exogenous SRIF application decreased rhythms generation (Pantaleo et al., 2011; Ramírez-Jarquín et al., 2012). This demonstrated that the peptide exerts a tonic inhibitory control on the rythmogenic neurons in order to avoid deleterious overactivity, probably through cellular subdomain-specific inhibitory and excitatory synaptic contacts (Wei et al., 2012). The existence of long-range somatostatinergic projections to either contralateral PreBötC (Stornetta et al., 2003) or downstream premotor neurons (Tan et al., 2010) favors a neuromodulatory role for PreBötC SRIF (Llona and Eugenín, 2005), whose developmental impairment may be involved in human pathologies (Schwarzacher et al., 2011) such as the sudden infant death syndrome (Lavezzi and Matturri, 2008).

## SOMATOSTATINERGIC NETWORKS IN PATHOLOGICAL CONDITIONS

In animals, an alteration of SRIF systems is observed during normal aging (Stanley et al., 2012) and pathological models of aging. In human a similar specific dysregulation is observed in normal pathological disorders such as some neurodegenerative and psychiatric diseases (Glorioso et al., 2011; Gleichmann et al., 2012).

### ALZHEIMER’S DISEASE

Somatostatin has been involved in Alzheimer’s disease (AD) pathology for a number of years. Indeed, since the early 1980s, it is known that SRIF levels in cortex and hippocampus are decreased in AD patients (Davies et al., 1980). Later, it was demonstrated that the decline in SRIF concentrations in the CSF (Tamminga et al., 1987) or in the middle frontal gyrus (Dournaud et al., 1995) correlates with cognitive deficits. Using quantitative real-time PCR, a recent study confirmed this decrease of SRIF in the inferior, medial, and superior temporal lobe of AD patients (Gahete et al., 2010). Interestingly, SRIF concentrations were reported to be significantly lower in Alzheimer patients carrying the epsilon 4 allele of APOE (Grouselle et al., 1998), the main genetic risk factor described to date for late-onset AD (Genin et al., 2011). In addition, two different studies found in Finnish and Chinese patients that polymorphisms in the SST gene are associated with the risk of developing AD (Vepsalainen et al., 2007; Xue et al., 2009).

Regarding SRIF receptors, data are limited and controversial. Although all studies agreed on a decrease of SRIF receptors in AD, controversies appeared about the proportion, the localization, and receptor subtype specificity of this decrease. SRIF receptor quantification using quantitative real-time PCR in AD temporal lobe showed a decrease of sst_1_, sst_3_, and sst_4_ receptors whereas sst_2_ and sst_5_ receptors were unchanged (Gahete et al., 2010). Previously, an immunohistochemistry study reported a similar decrease of sst_4_ but showed a reduction in neuronal sst_5_ – and a modest decrease in sst_2_ –like immunoreactivity without any changes in sst_1_ immunoreactive neurons (Kumar, 2005). Surprisingly, in the same study, an increase of sst_3_ subtype was observed in AD cortex. A radioligand binding and functional study showed a general receptor decrease in AD brain (Beal et al., 1985). More specifically, receptors levels in the frontal and temporal cortex were reduced by approximately 50% of control values in AD patients while a 40% reduction was reported in the hippocampus and no significant changes were found in the cingulate cortex, postcentral gyrus, temporal pole, and superior temporal gyrus. Another radioligand binding study revealed that while the maximal binding capacity of the SRIF-1 receptor subtype (primarily sst_2_, and possibly sst_5_) is altered in frontal and temporal cortices, other putative cortical SRIF receptor classes (SRIF-2 sites, i.e., sst_1_ and sst_4_) are not as broadly affected (Krantic et al., 1992). Finally, a last study showed a significant decrease only in the frontal cortex, but not in other brain regions (Bergstrom et al., 1991). Because of the cholinergic hypothesis regarding AD etiology, it was concluded that the pattern of change of SRIF binding in AD cortex might be secondary to the degeneration of SRIF receptor-bearing cholinergic afferents arising from the nucleus basalis. In line with this idea, experiments in the literature demonstrate that the selective destruction of cholinergic neurons of the basal forebrain with intracerebroventricular injection of 192-IgG saporin produces an irreversible loss of SRIF-immunoreactive neurons in the hilus of the hippocampus (Jolkkonen et al., 1997) and in the cortex (Zhang et al., 1998). This last study shows a correlation between the intensity of acetylcholinesterase in the cortex and the number of remaining SRIF cells. These data highlight a trophic dependence of SRIF neurons on cholinergic inputs and are consistent with observations in AD and aging.

Although SRIF deficit is not correlated with the amyloid load in AD brain patients (Dournaud et al., 1995), SRIF was identified as a modulator that increases brain neprilysin activity, one of the main enzymes involved in Aβ degradation (Saito et al., 2005). Recently, it has been shown that neuropeptide pituitary adenylate cyclase-activating polypeptide slows down AD-like pathology and improves cognition in a transgenic mouse model of AD through the activation of SRIF-neprilysin cascade (Rat et al., 2011). In mouse primary embryonic neurons, SRIF concomitantly increased neprilysin activity and decreased Aβ42 in the culture medium and these effects were blocked by an sst_5_ antagonist (but also an agonist at sst_1_ and sst_3_ receptors; for review, see Epelbaum et al., 2009). Moreover, neprilysin activity was decreased by 50% and Aβ42 increased by a similar extent in SRIF KO mice (Saito et al., 2005). Such findings may have important implications for understanding the cellular mechanisms leading to AD and suggest that SRIF and its receptors are potential pharmacological targets for AD. Indeed, FK962, which promotes SRIF production in the brain, co-administrated with donepezil, an acetylcholinesterase inhibitor widely used to treat patients, enhances cognition in rat and has been proposed as an add-on therapy for AD (McCarthy et al., 2011). In addition, Rubio et al. (2012) recently suggested that SRIF and CST act as a protective agent against Aβ toxicity. However, in APP transgenic mouse models, data concerning SRIF-containing interneurons are contradictory. In the triple-transgenic model of AD, 3×Tg-AD, inhibitory neurotransmission is unchanged in the cerebral cortex and hippocampus (Gleichmann et al., 2012). In a APP/PS1 mouse model of AD, as soon as 6 months of age, a decrease in the number of oriens-lacunosum moleculare hilar perforant path-associated SRIF-positive interneurons was evidenced in the hippocampus, when no change was demonstrated for 21 additional mRNA markers tested (Ramos et al., 2006). In the APPswe/PS1dE9 mouse model, Aβ deposition disrupted cognitive circuits when the cholinergic and somatostatinergic systems remained relatively intact (Savonenko et al., 2005). Another study on this last model even found that, in most brain regions tested, SRIF concentrations were increased rather than decreased relative to controls (Horgan et al., 2007). Thus, the validity of a direct and major role for SRIF in the regulation of Aβ42 degradation remains to be further confirmed (Iwata et al., 2005). More recent studies, focusing on olfaction, an early-altered function in AD (Wilson et al., 2009), account for evidence of a relationship between Aβ pathology and SRIF alterations in the disease. Indeed, SRIF interneurons and receptors are selectively reduced by approximately 50% in the anterior olfactory nucleus of AD patients (Saiz-Sanchez et al., 2010). These authors suggested that SRIF decreases in AD might be linked with Aβ. Moreover, an increase in the levels of aggregated Aβ peptide is observed with aging in olfactory cortices of APP/PS1 transgenic mouse model of AD, and it is accompanied by a fall in numbers of SRIF-positive interneurons (Saiz-Sanchez et al., 2012).

Experiments from our laboratory demonstrated that intrahippocampal injections of Aβ in rats induced aberrant inhibitory septo-hippocampal network activity associated with an impairment of hippocampal memory processes (Villette et al., 2010). This effect can be explained by the selective loss of long-range hippocampo-septal projecting neurons population containing calbindin and SRIF (Villette et al., 2012). This population of SRIF neurons could be a favored target for Aβ, explaining the early decrease of SRIF observed in AD.

Somatotropin release inhibiting factor is not only interacting with Aβ42 in AD, it has also an effect on Tau phosphorylation. Rubio et al. (2008) indicated that in mouse cortex SRIF and CST induce Tau phosphorylation at Ser262, a site modified in AD (Wang et al., 2007), although with different kinetics. An sst_2_/sst_4_ interaction seems implicated in this process but the types of phosphatases that are involved remain to be determined. Moreover, in human apoE4 knock-in mice where Tau phosphorylation and intracellular neurofibrillary tangle-like deposits are detected (Huang et al., 2001; Harris et al., 2003; Brecht et al., 2004), Huang’s group showed that the number of SRIF-positive interneurons correlated inversely with the performance of these mice in a spatial memory task (Andrews-Zwilling et al., 2010).

### PARKINSON’S DISEASE

Alteration of SRIF levels is also observed in other neurodegenerative diseases. Indeed, decrease in SRIF levels has been described in demented Parkinson’s disease patients (Epelbaum et al., 1989) as well as in a unilateral 6-OHDA experimental mouse model of Parkinson’s disease (Nilsson et al., 2009). Recent data obtained in a rat model of Parkinsonism showed that an alteration of presynaptic modulation by SRIF after dopamine deprivation. This observation may underlie a homeostatic mechanism trying to compensate for the excitability imbalance between direct and indirect basal ganglia pathways found during Parkinson’s disease (Lopez-Huerta et al., 2012).

### MAJOR DEPRESSIVE DISORDER

Evidence in major depressive disorder (MDD) suggests an impaired excitation/inhibition balance that is potentially mediated by decreased GABA content (Levinson et al., 2010). More specifically, Sibille et al. (2011) reported a down-regulation of SRIF in the dorsolateral prefrontal cortex (PFC), the subgenual cingulate cortices (Tripp et al., 2011), and the amygdala (Guilloux et al., 2011) of MDD patients. Engin et al. (2008a) and Engin and Treit (2009) revealed an antidepressant effect of SRIF mediated by either sst_2_ or sst_3_ receptor and suggested that while SRIF itself is not appropriate for clinical use because of its short half-life and diverse range of effects (Pinter et al., 2006), a closely related SRIF derivative may have some potential for the pharmacological treatment of depression.

### SCHIZOPHRENIA

One of the most consistent findings in schizophrenia neuropathology is deficits in cortical inhibitory interneurons across multiple cortical regions (Hashimoto et al., 2008). It has been known for years that cerebral cortical concentrations of SRIF are reduced in schizophrenics (Roberts et al., 1983) as well as hippocampal concentration (Ferrier et al., 1983; Konradi et al., 2011). Moreover, Hashimoto et al. (2008) found that subjects with schizophrenia exhibited deficits in SRIF expression in the PFC, and this was further confirmed after global analysis from six previously published microarray studies (Perez-Santiago et al., 2012). A recent study suggested that this decrease of SRIF-positive inhibitory interneurons in the PFC may be related to changes in an inflammatory response pathway that are often observed in schizophrenics (Fillman et al., 2012). In addition, Beneyto et al. (2012) showed that SRIF neurotransmission in the PFC of subjects with schizophrenia is also altered at the postsynaptic level in a receptor subtype-, layer-, and cell type-specific manner. The expression of sst_2_, but not sst_1_, mRNA is preferentially lower in layers 5–6, and in larger, putative pyramidal neurons in those layers. These authors suggested converging pre- and postsynaptic mechanisms to reduce inhibitory neurotransmission in pyramidal neurons in the PFC, which could alter the synchronization of low frequency oscillations and disturb working memory performance in subjects with schizophrenia.

### EPILEPSY

Somatostatin is highly expressed in brain regions associated with seizures and has been implicated as playing a prominent role in epilepsy (Vezzani and Hoyer, 1999) based on the observation of an activity-dependent release of SRIF during seizures, the modulation of SRIF mRNA expression, peptide and receptors levels by seizures and the effect of SRIF and its analogs on seizures (Tallent and Qiu, 2008; Zafar et al., 2012). Temporal lobe epilepsy (TLE) is characterized by hippocampal sclerosis together with profound phenotypic changes of different classes of interneurons. Hilar SRIF interneurons undergo extensive degeneration in patients with hippocampal sclerosis (de Lanerolle et al., 1989; Robbins et al., 1991). Recently, this selective neurodegeneration has been linked to the specific enrichment of somatostatinergic neurons in striatum-enriched phosphatase, an enzyme that counteracts the MAPK neuroprotective pathway (Choi et al., 2007; Florio et al., 2008). SRIF receptors may represent potential therapeutic targets for TLE. Indeed, SRIF is released in characteristic conditions of seizures and SRIF and its analogs affect seizures (Vezzani and Hoyer, 1999; Buckmaster et al., 2002). However, information on the precise contribution of each SRIF receptor on the SRIF-induced inhibition of epileptiform activity is still limited. Although the sst_2_ receptor is likely to mediate the anticonvulsant effects of SRIF in rat hippocampus (Vezzani and Hoyer, 1999), observations in the mouse support a central role for sst_4_ (Moneta et al., 2002) and/or sst_1_ receptors (Cammalleri et al., 2004, 2006) in mediating SRIF inhibition of epileptiform activity. In a rodent model of cortical focal ischemia, sst_2_ is also activated while the infarct size is significantly reduced in sst_2_ KO mice (Stumm et al., 2004). However, recent data in rats showed that sst_1_ receptors do not appear to mediate the *in vivo* anticonvulsive effect of SRIF (de Bundel et al., 2010), whereas sst_3_ and sst_4_ mediate this effect through a functional interaction with sst_2_ receptor (Aourz et al., 2011).

## CONCLUSION

Somatostatin systems are widely expressed in the different brain regions and are involved in numerous processes from sensory to cognitive functions, suggesting that they play major roles in brain functioning. These key roles are illustrated by the decrease of SRIF concentrations observed in neurodegenerative diseases such as AD and Parkinson’s disease but also in psychiatric diseases such as schizophrenia and MDD. From this perspective, SRIF systems represent a potential and challenging therapeutic target. Further studies need to be carried on to unravel the role of SRIF systems in all functions they have been implicated in.

## Conflict of Interest Statement

The authors declare that the research was conducted in the absence of any commercial or financial relationships that could be construed as a potential conflict of interest.
